# Nabe: an energetic database of amino acid mutations in protein–nucleic acid binding interfaces

**DOI:** 10.1093/database/baab050

**Published:** 2021-08-14

**Authors:** Junyi Liu, Siyu Liu, Chenzhe Liu, Yaping Zhang, Yuliang Pan, Zixiang Wang, Jiacheng Wang, Ting Wen, Lei Deng

**Affiliations:** School of Computer Science and Engineering, Central South University, 22 Shaoshan South Road, Changsha 410075, China; Viterbi School of Engineering, University of Southern California, 3650 McClintock Ave. OHE 106, Los Angeles, CA 90089, USA; School of Computer Science and Engineering, Central South University, 22 Shaoshan South Road, Changsha 410075, China; School of Computer Science and Engineering, Central South University, 22 Shaoshan South Road, Changsha 410075, China; School of Computer Science and Engineering, Central South University, 22 Shaoshan South Road, Changsha 410075, China; School of Computer Science and Engineering, Central South University, 22 Shaoshan South Road, Changsha 410075, China; School of Computer Science and Engineering, Central South University, 22 Shaoshan South Road, Changsha 410075, China; School of Computer Science and Engineering, Central South University, 22 Shaoshan South Road, Changsha 410075, China; School of Computer Science and Engineering, Central South University, 22 Shaoshan South Road, Changsha 410075, China; School of Computer Science and Engineering, Central South University, 22 Shaoshan South Road, Changsha 410075, China

## Abstract

Protein–nucleic acid complexes play essential roles in regulating transcription, translation, DNA replication, repair and recombination, RNA processing and translocation. Site-directed mutagenesis has been extremely useful in understanding the principles of protein–DNA and protein–RNA interactions, and experimentally determined mutagenesis data are prerequisites for designing effective algorithms for predicting the binding affinity change upon mutation. However, a vital challenge in this area is the lack of sufficient public experimentally recognized mutation data, which leads to difficulties in developing computational prediction methods. In this article, we present Nabe, an integrated database of amino acid mutations and their effects on the binding free energy in protein–DNA and protein–RNA interactions for which binding affinities have been experimentally determined. Compared with existing databases and data sets, Nabe is the largest protein–nucleic acid mutation database, containing 2506 mutations in 473 protein–DNA and protein–RNA complexes, and of that 1751 are alanine mutations in 405 protein–nucleic acid complexes. For researchers to conveniently utilize the data, Nabe assembles protein–DNA and protein–RNA benchmark databases by adopting the data-processing procedures in the majority of models. To further facilitate users to query data, Nabe provides a searchable and graphical web page.

**Database URL**: http://nabe.denglab.org

## Introduction

Protein–nucleic acid interactions play vital roles in many biological cell activities, including transcription, translation, DNA repair, metabolic regulation and immune recognition (1, 2). Given the paramount impact that protein–nucleic acid interactions have on cellular processes, the single amino acid substitution that happened in the proteins may cause severe perturbations or complete loss of function, potentially leading to diseases, like cancers (3) or neurodegenerative diseases. For example, the L351P mutation is identified in the RNA-binding motif protein 28, causing alopecia neurologic defects and endocrinopathy syndrome. Mutation D169G in the transactive response (TAR) DNA-binding protein causes the aberrant function of the protein, leading to amyotrophic lateral sclerosis type 10 (4). Therefore, determining functionally crucial missense mutations may provide a clue to interpreting the pathology at the molecular level and expediting the development of their treatment and prevention.

With a huge stride made in biological technology, one feasible approach to quantify the influence of a mutant on protein–nucleic acid interactions is to experimentally determine the binding free energy change using site-directed mutagenesis methods, such as surface plasmon resonance (5), isothermal titration calorimetry (6) and fluorescence resonance energy transfer (FRET) (7). However, these biological experiments are so time-consuming and costly that plenty of researchers turn to computational methods for novel insights. Peng *et al.* developed SAMPDI (8) to predict the protein–DNA binding affinity change about single amino acid alterations based on modified Molecular Mechanics/Poisson-Boltzmann Surface Area (MM/PBSA) along with supplementary knowledge-based terms derived from the physicochemical attributes of protein–DNA complexes. Pires *et al.* presented a scalable method, named mCSM-NA (9), to predict and identify the effect of a single-point missense mutation on protein–nucleic acid binding, relying on graph-based signatures. Zhang *et al.* proposed PremPDI (10), a model based on molecular mechanics force fields and fast side-chain optimization algorithms to evaluate the quantitative change in protein–DNA binding affinity upon single-sequence variants. Although these three methods have attained far-reaching impact on the field of analyzing variations in the protein–nucleic acid complexes, there are still obstacles that hinder further advancement, and the dominant handicap is the deficiency of data quality and quantity. For one thing, the data quality in the methods cannot be guaranteed because the data mainly come from separated references and the ProNIT database (11, 12), an outdated thermodynamic database of protein–nucleic acid interactions constructed by Prabakaran *et al.*, and could not meet current research requirement. For another thing, the average data volume at present is around 200 mutations and too insufficient to keep up with the trend of artificial intelligence. The inadequacy of data makes it arduous to apply machine learning to this realm and perform its fabulous power. Consequently, in view of the above two concerns, it is urgent to develop a comprehensive and up-to-date database for the single amino acid substitutions in protein–nucleic acid complexes.

In addition, among total variations, residues that mutate into alanine have been given special attention because of their contribution to pinpointing hot spots in the protein–nucleic acid complexes. Hot spots refer to a small portion of residues that devote most of the binding free energy in the interaction (13). Through the alanine scanning mutagenic experiments (14), researchers are able to accurately tag hot spots and further probe deeper about the priority of them in cellular progressions. For example, during the formation of tumors, the p53 protein, a transcription factor, serves a necessary function as a suppressor (15). Thus, the point mutations in the p53 protein would not only invalidate tumor suppressor functions in cell cycle arrest and apoptosis but also confer novel oncogenic functions (16). More importantly, amidst all the recognized cancer-related p53 mutations, over 80% are located within the core domain, where six hot spots (Arg-175, Gly-245, Arg-248, Arg-249, Arg-273 and Arg-282) account for about 40% of whole mutations (17), indicating that hot spot mutp53 proteins, such as mutp53GOFs, are more likely to induce insensitivity to drugs, resistance to apoptosis, enhanced cell proliferation and/or migration, increased chromosomal instability and non-homologous recombination. Except for this example, emerging evidences have shown that hot spots are essential in the molecular recognition mechanisms and regulation and also form a solid foundation for bioengineering, such as protein engineering and drug design (18).

Thus, ample researchers are attracted to utilize computational methods for the prediction of hot spots complementing for the expensive cost of experimental methods. Xiaolei Zhu *et al.* built a knowledge-based model named iPNHOT (19) to predict the hot spots on protein–nucleic acid interfaces with a two-step feature selection strategy. For protein–DNA complexes, Yuliang Pan *et al.* proposed PreHots (20) for predicting hot spots, which adopts an ensemble stacking classifier with 19 features selected by a sequential backward feature selection algorithm. Zhang *et al.* developed a feature-based method, termed PrPDH (21), to predict the hot spots in protein–DNA binding interfaces, employing SVM based on the 10 optimal features selected by random forests (VSURF) algorithm. Ke Li *et al.* presented a computational model, namely sxPDH (22), based on supervised isometric feature mapping (S-ISOMAP) and extreme gradient boosting (XGBoost) for more accurate predictions. Moreover, for the protein–RNA complexes, Zhang *et al.* introduced a novel sequence-based method, called SPHot (23), that integrates an ensemble classifier to predict hot spots. Yuliang Pan *et al.* created the PrabHot (24) model that uses the Boruta algorithm to select features and the ensemble vote classifier (EVC), including GTB, SVM and ERT classifiers, to give the eventual results of the hot spot prediction. Deng *et al.* provided the XGBPRH (25) method based on an XGBoost algorithm and an optimal set of properties extracted by a two-step feature selection algorithm, containing Max-Relevance and Min-Redundancy (mRMR) and sequential forward feature selection algorithm. Although the researchers have achieved some accomplishments in this area, compared with the matured prediction of hot spots on protein–protein interfaces (26–28), the study of hot spots in protein–nucleic acid is still at the developing stage in terms of the amount of related papers and the application of state-of-art machine learning methods. The primary reason why the exploring progress of protein–nucleic acid hot spots falls behind that of protein–protein hot spots is the paucity of organized alanine mutagenic databases for protein–nucleic acid complexes. For instance, there are various protein–protein thermodynamic hot spot databases, such as Alanine Scanning Energetics Database (29), Binding Interface Database (30), SKEMPI database (31), Assi *et al.*‘s Ab+ data (32) and Petukh *et al.*’s Alexov_sDB (33). However, currently, there is only a handful of protein–nucleic acid hot spot databases, like the database of alanine mutagenized protein–nucleic acid interactions (dbAMEPNI) (34) constructed by Ling Liu *et al.*, which contains limited data and stopped updating in 2017. Moreover, the data of hot spots face ever severe issues mentioned in the mutants of protein–nucleic acids. In terms of data quantity, the data collection range of hot spots is relatively narrow because the scope is limited to alanine mutation. As for the data quality, apart from the problem of outdated data, each method has a separate data set and uses inconsistent data format, as well as the data-processing procedures. Accordingly, it is imperative to methodically organize the alanine mutant protein–nucleic acid data.

In this article, we present a systematic database, the protein–nucleic acid binding energy database (Nabe Database), to address the aforementioned data plight encountered in the field of protein and nucleic acid mutation. Nabe database is an energetic database of amino acid mutations in protein–nucleic acid binding interfaces and centers on the hot spot data, offering independent subsets. With respect to data volume, Nabe is the largest protein–nucleic acid mutation database until now, containing more than 2500 mutations in more than 470 protein–DNA and protein–RNA complexes, supplemented with experimental conditions, literature, structural and functional information, and links to other databases for protein sequence, structure, and interaction network. Furthermore, in regard to data quality, Nabe has amassed entire mutation data since 2020, ensuring that the data within the database is up to date and unifies the data format with that used by the majority of methods. In order for researchers to relatively fairly compare the performance between their methods and other advanced methods on the identical data set, Nabe assembles protein–DNA and protein–RNA benchmark databases through adopting the data-processing procedures in most models. To further facilitate users to query data, Nabe provides a searchable and graphical web page, which is available at http://nabe.denglab.org.

## Results and discussion

### Data acquisition and analysis

**Figure 1.**
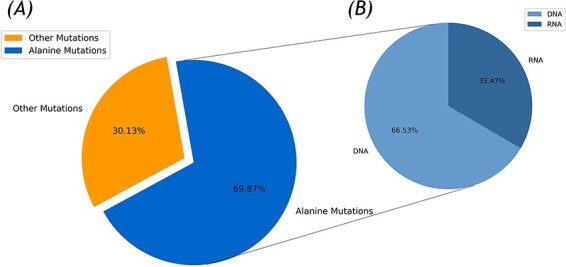
Statistics of the Nabe database. (A) Alanine mutation percentage chart. (B) The ratio of DNA to RNA in alanine mutation.

In the current version, 336 protein–DNA and 137 protein–RNA complexes were collected for the Nabe database by the integration of the Protein Data Bank (PDB) entries of the protein–nucleic acid in the PDB database with over 500 corresponding papers for a total of 2506 mutants. Due to the disparate experimental environments and multiple binding affinity measurement methods, the same mutant has several unequal ∆∆G values and that was also recorded in the Nabe database. Each such record is differentiated by different temperature value or reference id and the experimental details could be found through linked references. As illustrated in Figure 1A, among the 2506 mutants, there are 755 other mutants and 1751 alanine mutations, accounting for more than two-thirds of the total. These high proportion of alanine mutations are aimed to pinpoint hot spots in protein–nucleic acid binding interfaces and further accelerate the researching progress of protein–nucleic acid interaction. Additionally, from Figure 1B, the whole alanine variants are from 405 protein–nucleic acid complexes, of which 275 are protein–DNA complexes (containing 1165 alanine variants in DNA) and 130 are protein–RNA complexes (including 586 alanine variants in RNA).

**Figure 2.**
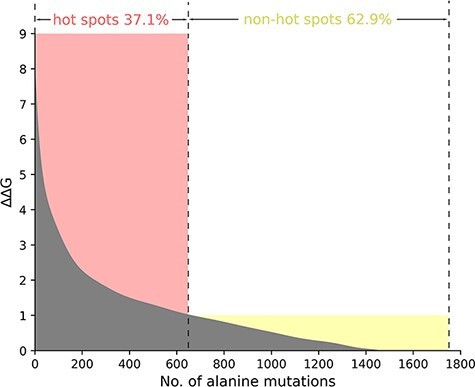
The ΔΔG range of hot spots (red, ΔΔG >1.0 kcal/mol) and non-hot spots (yellow, ΔΔG <1.0 kcal/mol) is indicated. The section where ΔΔG is less than 0 is excluded in the picture.

Taking into account the standard definition of hot spots in the vast majority of the protein–nucleic acid hot spot prediction literature, we define the residues where ∆∆G value is more than 1.0 kcal/mol as hot spots. Thus, in the total 1751 alanine variants, there are 650 hot spots and 1101 non-hot spots as demonstrated in Figure 2. In Figure 2, we sort and number the mutants in descending order according to their ∆∆G value. We divide the whole figure into two parts with the definition of hot spots.

**Figure 3.**
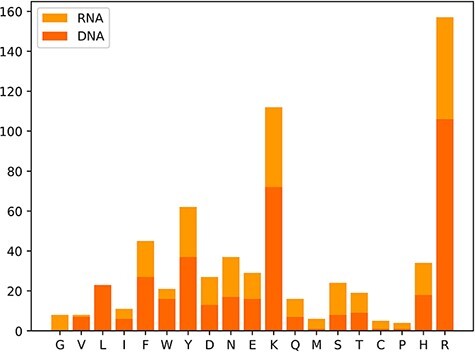
Distribution map of amino acid residues in hot spots.

We also classify these 650 hot spots into two types: 385 hot spots related to DNA and 265 hot spots associated with RNA, which are derived from a total of 232 protein–nucleic acid complexes, including 144 protein–DNA complexes and 88 protein–RNA complexes. Moreover, we observe the amino acid distribution in the hot residues, which is depicted in Figure 3. In Figure 3, the x-axis represents the abbreviations of 19 amino acids except alanine, and the y-axis represents the number of hot residues, and the DNA/RNA hot residues are counted, respectively. Notably, the top three amino acid residues most possibly to be hot spots are arginine (R), lysine (K) and tyrosine (Y). The analysis of the amino acid composition in hot spots illustrates a clear preference for lysine (K) and arginine (R) with a sum occurrence of nearly 40% of the whole.

In addition to the original data, we also perform the data-processing procedures mentioned in the ‘Materials and methods’ to gain two benchmark databases for DNA and RNA, respectively. The DNA benchmark subset encompasses 664 alanine mutants from 160 protein–DNA complexes, containing 254 hot spots and 410 non-hot spots. The RNA benchmark subset consists of 400 alanine mutations from 90 protein–RNA complexes, including 180 hot spots and 220 non-hot spots with the ratio close to 1:1. The two benchmark data sets are currently the largest collections under the same data-processing method. Specialists who focus on the prediction of hot spots in protein–nucleic acids are capable of using the benchmark databases for training models directly and then deliver impartial outcomes under the same standard data sets.

### Comparison with existing databases

In order to further verify the comprehensiveness of the Nabe database, we select present-day databases and data sets gleaned from multifarious models for comparison. For a clear visualization of the comparison, we separate the database and the data sets and further subdivide the data sets into two sections according to their methods’ focus. Table 1 manifests the comparison between the Nabe database and other databases in the amount of mutation data and the timeliness of data. The ‘Year’ column in the Table 1 refers to the time when the database was last updated. As revealed in Table 1, the mutation data of the Nabe database is more than four times that of dbAMEPNI and almost twice that of the ProNIT database. Besides, the complexes stored in the Nabe database exceed the sum of the complexes in the other two databases. Overall, the Nabe database is the currently largest database of amino acid mutagenic effects for protein–nucleic acid interactions.

**Table 1.** Comparison with other thermodynamic protein–nucleic acid databases

Name	Mutations	Complexes	Year	Reference(s)
ProNIT	1411	158	2005	11, 12
dbAMEPNI	577	152	2017	34
Nabe	2506	473	2020	This article

For those data sets, because the data sets of several methods insert novel data collected by themselves that do not appear in the above two databases, we divide this part of the data sets into an independent category. For the sake of assuring the timeliness of the data, we only opt for methods in the past 3 years. Besides, the SAMPDI, PremPDI and mCSM-NA methods are aimed to examine the impact of all amino acid mutations on protein–nucleic acid interactions (as illustrated in Table 2), while the remaining other methods only fixate on alanine mutations and hot spots (as shown in Table 3). Therefore, we further refine the data sets into the following two tables. For each table, to reach a reasonable standard, we add an extra column ‘Type’ to distinguish whether the partner that the methods concentrate on is DNA or RNA. Table 2 proves that the data in the Nabe database is more than seven times that of other data sets, regardless of whether the partner is DNA or the total of DNA and RNA. That again confirms the fact that Nabe is the database with the most extensive data compilation.

**Table 2.** The comparison with the data sets extracted from methods of predicting the changes in protein–nucleic acid binding affinity caused by amino acid mutations

Name	Type	Mutations	Complexes	Year	Reference
SAMPDI	DNA	105	13	2018	(8)
PremPDI	DNA	219	49	2018	(10)
Nabe	DNA	**1721**	**336**	**2020**	This article
mCSM-NA	DNA/RNA	331	38	2017	(9)
Nabe	DNA/RNA	**2506**	**473**	**2020**	This article

**Table 3.** The comparison with the original data sets without data processing extracted from methods of predicting protein–nucleic acid hot spots

Name	Type	Mutations	Complexes	Year	Reference
PreHots	DNA	660	162	2020	(20)
PrPDH	DNA	414	108	2020	(21)
sxPDH	DNA	414	108	2020	(22)
Nabe	DNA	**1165**	**275**	2020	This article
SPHot	RNA	350	63	2019	(23)
PrabHot	RNA	350	63	2017	(24)
XGBPRH	RNA	350	63	2019	(25)
Nabe	RNA	**586**	**130**	**2020**	This article
iPNHOT	DNA/RNA	417	137	2020	(19)
Nabe	DNA/RNA	**1751**	**405**	2020	This article

In Table 3, on the grounds that distinct models have slight differences in the way data is processed, we uniformly take unprocessed raw data for comparison. Whether the type is DNA or RNA, the data in the Nabe database is nearly double the data in the listed data sets. Apart from comparing with the row data, we also select several data sets with the same data-processing method for comparison. For the protein–DNA complexes, PrPDH and sxPDH use the same benchmark data set with 214 mutations and 64 complexes, and PreHots obtains a benchmark data set with 260 mutations and 89 complexes. However, Nabe provides a protein–DNA benchmark database with 664 mutations and 160 complexes, which is near twice the largest of the two benchmark data sets. For the protein–RNA complexes, the three listed data sets all use the same benchmark database generated by PrabHot with 209 mutations and 47 complexes. Nevertheless, the RNA benchmark data set in the Nabe database is approximately twice the PrabHot’s benchmark data set. In conclusion, the Nabe database is currently the largest database for the research of the hot spots in the protein–nucleic acid complexes and the mutagenic impact on the protein–nucleic acid interactions. It is worth noting that Nabe will be beneficial in improving the functionality and accuracy of existing prediction methods.

### Website interface

**Figure 4.**
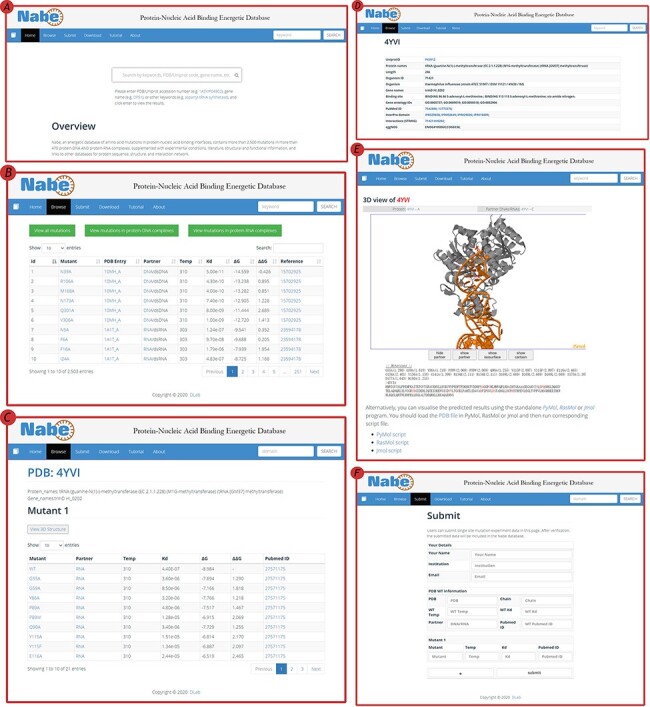
User interface of the Nabe database. (A) The Home page with a quick search box. (B) The Browse web page. (C) The mutants page of a protein–nucleic acid complex. (D) The detail page of a protein–nucleic acid complex. (E) The 3D model of a protein–nucleic acid complex. (F) The Submit web page.

The Nabe database provides a variety of web-based interfaces and graphical visualizations to conveniently search and analyze protein–nucleic acid interaction data in databases (Figure 4). At the ‘Home’ page (Figure 4A), users are able to type keywords (e.g. aspartyl-tRNA synthetase), PDB/Uniprot codes (e.g. 1ASY / P04802) or gene names (e.g. *DPS1*), etc. in the search box to query the results. The server will return the result of the matched fuzzy query information, including the PDB ID, protein name, Uniprot ID, gene name and organism, and the user can select one of the complexes to view the mutagenized data.

On the ‘Browse’ page (Figure 4B), users could straightforwardly view all the residue information in the database or separately classified protein–DNA/protein–RNA data. The ‘Browse’ page displays the mutagenesis experiment information of each residue, including temp, Kd, ∆G, ∆∆G and reference. There is also a quick search window at the upper right corner for users to retrieve the needed residue items For each PDB entry, users would scrutinize entire variants in the mutants page (Figure 4C) and also look through detailed information about proteins and binding nucleic acids (Figure 4D), including uniprot id, protein name, organism, gene name, binding site, gene ontology IDs, pubmed id, interpro domain, interaction (string) and eggnog. In order to obviously uncover the features of the data, the Nabe database is equipped with 3D models of the protein–nucleic acid complexes (Figure 4E), listing the ∆∆G score for each residue at the bottom and providing an interactive operation that allows the mutated data to be more closely integrated with the visualization. We also provide users with a tree view format to choose.

The Nabe database also holds a ‘Submit’ page (Figure 4F) that encourages users to submit novel data to the database and welcomes more scientists to engage in the protein–nucleic acid field to form a more prosperous community. At the ‘Submit’ page, users would fill in the form about the new PDB entry and the corresponding mutants. Once verified, the submitted data will be added to the Nabe database. We will send a confirmation email to the users’ mailboxes.

Through the ‘Download’ page, users have access to all provided mutagenized data files. On this page, four data sets are listed, including the whole mutagenesis data, the alanine mutagenesis data and the two DNA and RNA benchmark data sets. Information about the data format and its explanation are also specified under each data set. Besides, with the supplementary benchmark data sets, scientists will save considerable time dealing with data and throw themselves into the model building and optimization.

The ‘Tutorial’ page provides a tutorial of the operation available in the Nabe database and a meticulous description of each action. Moreover, there are detailed API usage instructions on the page. The Nabe API could be accessed with simple HTTP requests from any tool or programming language and allows users to retrieve a filtered list of mutants using the json format. Users may also use advanced functions of adding parameters to the requested query string to ignore irrelevant data. The ‘About’ page is also constructed, supplying a concise introduction and contact information. Overall, the Nabe website possesses uncomplicated operations and delightful interfaces for users at any technical level to seek out and utilize protein–nucleic acid mutated data.

## Conclusion

The current Nabe database version contains 336 protein–DNA and 137 protein–RNA complexes with a total of 2506 mutations, of which 1751 mutations are favorable for hot spot prediction in the protein–nucleic acid interfaces. The Nabe database is presently the largest and newest protein–nucleic acid mutations and hot spot database. The two benchmark data sets are also provided in the Nabe for handy usage. Besides, Nabe divides data in accordance with the category of the research focus and the type of protein–nucleic acid complexes to facilitate users to quickly locate the information they need. The thorough data accumulated in the Nabe database would definitely pave the way to promoting the prosperity of the field of protein–nucleic acid interaction.

In the future, we will continuously expand the database every 6 months. Our next step will be toward attaching the results of feature calculation to each mutation, such as network features, exposure features, sequence features and structure features. Then, we will further integrate cutting-edge models and our original methods for users to obtain a set of prediction outcomes in one stop.

## Materials and methods

### Data collection and compilation

**Figure 5.**
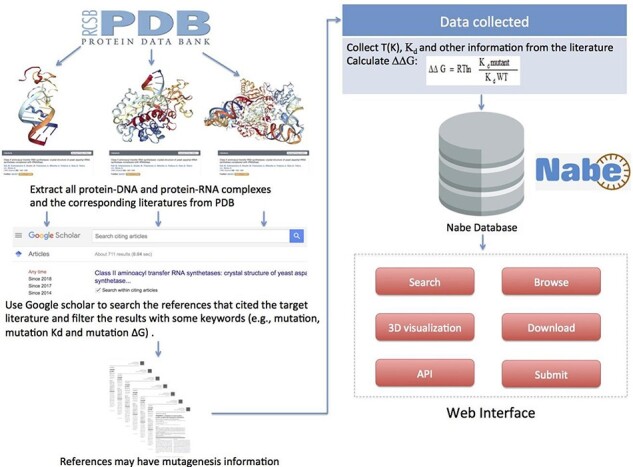
Flowchart describing data collection, workflow and applications of Nabe database.

To gather and compile data as comprehensively as possible, we collect mutagenized data for the Nabe database in the following four steps: (i) Initially, we cluster all the PDB entries of protein–nucleic acid complexes from the PDB database. (ii) For each PDB entry, we extract from the PDB database the corresponding literature that is the first one to depict the information about the complex in detail. (iii) Then we use the corresponding literature as the beginning points to search for ample references concerning mutation experiments because the subsequent measurements of binding free energy generally would cite the initial articles with sequence and structural information of the corresponding complexes. Specifically, we use Google Scholars to locate all the literature that cite the original ones and filter the results with some keywords (such as mutations, mutation Kd and mutation ∆G) to limit the scope of the query. (iv) Finally, we review each paper to manually extract experimental mutagenesis data and calculate the binding free energy change. Our focus is mainly on the method section, result section, tables and charts of every article and supplementary material. In addition, as we observe that most protein–nucleic acid hot spot prediction methods construct their data sets by merging different databases or downloading data from former models, we incorporate similar databases and data sets, such as dbAMEPNI and ProNIT, into the Nabe database to further expand the data volume and, more significantly, help researchers save time in gathering data. We first filter out duplicate data when compared with our database, and then manually check the validity of each record when adding it into the database. Overall, every record in the Nabe database is supported by a published reference in the PubMed or Google scholar, and each contains these 10 types of information: mutant, PDB entry, patterner, Kd value, ∆G, ∆∆G, experimental conditions, literature, structural and functional information of the complex and links to other databases, like PDB and Uniprot. The above collecting data procedures are plainly described in Figure 5.

Because we lay more eyes on hot spots, we separate the data of alanine mutations from other mutations, setting a new subset. We also add hot spot benchmark databases into the Nabe. For the benchmark data sets, we mainly concentrate on data preprocessing methods and removing the duplicate and non-interface residues of the mutation. The two non-redundant energetic benchmark data sets for protein–DNA and protein–RNA binding hot spots are established by two procedures that are commonly used in hot spot prediction models. To remove the redundancy, proteins with sequence similarity >40% were excluded by using Cluster Database at High Identity with Tolerance (CD-HIT). The interface residues were calculated based on the buried solvent-accessible surface area upon complex formation (∆ASA > 1 A ) and the relative solvent-accessible surface area (>5%) by using Naccess.

### Database architecture and web interface

We then integrate the collected data into MySQL, a relational database management system. Besides, for convenient access to the mutation data, we also design a user-friendly web interface, which is realized by Perl and Javascript. For a direct expression, we apply JSmol to visualize the mutants, which vividly displays the structure of the mutants instead of an obscure string. In summary, through the website, researchers will no longer be restricted by digital devices or the technology to browse and analyze data.

### Update prospect

The Nabe database welcomes all the biochemists and bioinformaticians to submit new published protein–nucleic acid mutagenized data through the ‘Submit’ page (Figure 4F) by filling in the form available on the website. Each data would be examined by our team and be included into the database after the verification. In addition, we will always pay close attention to the novel published experimental mutation data and update the database every 6 months in the future. For every update, we will not only enlarge the amount of data but also add diverse functions, like providing computational binding free energy prediction data, to form exhaustive integrity.

## References

[1] Pan X., Peter R., Yan J. et al. (2018) Prediction of RNA-protein sequence and structure binding preferences using deep convolutional and recurrent neural networks. *BMC**Genomics.*, 19, 511. 10.1186/s12864-018-4889-1 PMC6029131 29970003

[2] Zeng J., Li D., Wu Y. et al. (2016) An empirical study of features fusion techniques for protein–protein interaction prediction. *Curr. Bioinform.*, 11, 4–12.

[3] Bobak K. and Janga S.C. (2014) Dissecting the expression landscape of RNA-binding proteins in human cancers. *Genome Biol.*, 15, 1–14. 10.1186/gb-2014-15-1-r14 PMC4053825 24410894

[4] Castello A., Fischer B., Hentze M.W. et al. (2013) RNA-binding proteins in Mendelian disease. *Trends Genet.*, 29, 318–327. 23415593 10.1016/j.tig.2013.01.004

[5] Teh H.F., Peh W.Y.X., Su X. et al. (2007) Characterization of protein–DNA interactions using surface plasmon resonance spectroscopy with various assay schemes. *Biochemistry*, 46, 2127–2135. 17266332 10.1021/bi061903t

[6] Velázquez-Campoy A., Ohtaka H., Nezami A. et al. (2005) *Isothermal Titration Calorimetry*. Humana Press, Clifton, New Jersey, United States.

[7] Hillisch A., Lorenz M. and Diekmann S. (2001) Recent advances in FRET: distance determination in protein–DNA complexes. *Curr. Opin. Struct. Biol.*, 11, 201–207. 11297928 10.1016/s0959-440x(00)00190-1

[8] Peng Y., Sun L., Zhe J. et al. (2017) Predicting protein–DNA binding free energy change upon missense mutations using modified MM/PBSA approach: SAMPDI webserver. *Bioinformatics*, 35, 779–786. 10.1093/bioinformatics/btx698 PMC6048991 29091991

[9] Pires D.E.V. and Ascher D.B. (2017) mCSM–NA: predicting the effects of mutations on protein–nucleic acids interactions. *Nucleic Acids Res.*, 45, W241–W246. 28383703 10.1093/nar/gkx236 PMC5570212

[10] Ning Z., Yuting C., Feiyang Z. et al. (2018) PremPDI estimates and interprets the effects of missense mutations on protein-DNA interactions. *PLoS Comput. Biol.*, 14, 12. 10.1371/journal.pcbi.1006615 PMC6303081 30533007

[11] Shaji K.M.D., Abdulla B.K., Michael G.M. et al. (2006) ProTherm and ProNIT: thermodynamic databases for proteins and protein–nucleic acid interactions. *Nucleic Acids Res.*, 34, 204–206. 10.1093/nar/gkj103 PMC1347465 16381846

[12] Prabakaran P., An J., Gromiha M.M. et al. (2001) Thermodynamic database for protein-nucleic acid interactions (ProNIT). *Bioinformatics*, 17, 1027–1034. 11724731 10.1093/bioinformatics/17.11.1027

[13] Irina S., Moreira P., Fernandes A. et al. (2007) Hot spots-A review of the protein-protein interface determinant amino-acid residues. *Proteins Struct. Funct. Bioinform.*, 68, 803–812. 10.1002/prot.21396 17546660

[14] Kortemme T., Kim D.E. and Baker D. (2004) Computational alanine scanning of protein-protein interfaces. *Sci. STKE Signal Transduction Knowl. Environ.*, 2004, pl2. 10.1126/stke.2192004pl2 14872095

[15] Baugh E.H., Ke H., Levine A.J. et al. (2017) Why are there hotspot mutations in the TP53 gene in human cancers? *Cell Death Differ.*, 25, 154–160. 29099487 10.1038/cdd.2017.180 PMC5729536

[16] Muller P.A.J. and Vousden K.H. p53 mutations in cancer. *Nat. Cell Biol.*, 15, 2–8. 23263379 10.1038/ncb2641

[17] Olivier M., Eeles R., Hollstein M. et al. (2002) The IARC TP53 database: new online mutation analysis and recommendations to users. *Hum. Mutat.*, 19, 607–614. 12007217 10.1002/humu.10081

[18] Xia J., Yue Z., Di Y. et al. (2016) Predicting hot spots in protein interfaces based on protrusion index, pseudo hydrophobicity and electron-ion interaction pseudopotential features. *Oncotarget*, 7, 1–11. 26934646 10.18632/oncotarget.7695 PMC4951271

[19] Zhu X., Liu L., He J. et al. (2020) iPNHOT: a knowledge-based approach for identifying protein-nucleic acid interaction hot spots. *BMC Bioinform.*, 21, 1–24. 10.1186/s12859-020-03636-w PMC7336410 32631222

[20] Pan Y., Zhou S. and Guan J. (2020) Computationally identifying hot spots in protein-DNA binding interfaces using an ensemble approach. *BMC Bioinform.*, 21, 384. 10.1186/s12859-020-03675-3 PMC7495898 32938375

[21] Zhang S., Zhao L., Zheng C.-H. et al. (2020) A feature-based approach to predict hot spots in protein–DNA binding interfaces. *Brief. Bioinform.*, 21, 1038–1046. 30957840 10.1093/bib/bbz037

[22] Li K., Zhang S., Yan D. et al. (2020) Prediction of hot spots in protein—DNA binding interfaces based on supervised isometric feature mapping and extreme gradient boosting. *BMC Bioinform.*, 21, 381. 10.1186/s12859-020-03683-3 PMC7495874 32938395

[23] Zhang S., Zhao L. and Xia J. (2019) SPHot: prediction of hot spots in protein-RNA complexes by protein sequence information and ensemble classifier. *IEEE Access*, 7, 104941–104946.

[24] Pan Y., Wang Z., Zhan W. et al. (2018) Computational identification of binding energy hot spots in protein–RNA complexes using an ensemble approach. *Bioinformatics*, 34, 1473–1480. 29281004 10.1093/bioinformatics/btx822

[25] Deng L., Sui Y. and Zhang J. (2019) XGBPRH: prediction of binding hot spots at protein–RNA interfaces utilizing extreme gradient boosting. *Genes*, 10, 1–14. 10.3390/genes10030242 PMC6471955 30901953

[26] Andrew A., Bogan A., Kurt S. et al. (1998) Anatomy of hot spots in protein interfaces - ScienceDirect. *J. Mol. Biol.*, 280, 1–9. 9653027 10.1006/jmbi.1998.1843

[27] Deng L., Guan J., Wei X. et al. (2013) Boosting prediction performance of protein-protein interaction hot spots by using structural neighborhood properties. *J. Computat. Biol.*, 20, 878–891. 10.1089/cmb.2013.0083 PMC3822376 24134392

[28] Siyu L., Chuyao L. and Deng L. (2018) Machine learning approaches for protein? Protein interaction hot spot prediction: progress and comparative assessment. *Molecules (Basel, Switzerland)*, 23, 2535–2545. 10.3390/molecules23102535 PMC6222875 30287797

[29] Thorn K.S. and Bogan A.A. (2001) ASEdb: a database of alanine mutations and their effects on the free energy of binding in protein interactions. *Bioinformatics*, 17, 284–285. 11294795 10.1093/bioinformatics/17.3.284

[30] Fischer T.B., Arunachalam K.V., Bailey D. et al. (2003) The binding interface database (BID): a compilation of amino acid hot spots in protein interfaces. *Bioinformatics*, 19, 1453–1454. 12874065 10.1093/bioinformatics/btg163

[31] Moal I.H. and Fernández-Recio J. (2012) SKEMPI: a Structural Kinetic and Energetic database of Mutant Protein Interactions and its use in empirical models. *Bioinformatics*, 28, 2600–2607. 22859501 10.1093/bioinformatics/bts489

[32] Assi S.A., Tanaka T., Rabbitts T.H. et al. (2010) PCRPi: Presaging Critical Residues in Protein interfaces, a new computational tool to chart hot spots in protein interfaces. *Nucleic Acids Res.*, 38, e86. 10.1093/nar/gkp1158 PMC2847225 20008102

[33] Petukh M., Li M. and Alexov E. (2015) Predicting binding free energy change caused by point mutations with knowledge-modified MM/PBSA method. *PLoS Comput. Biol.*, 11, 1–23. 10.1371/journal.pcbi.1004276 PMC4492929 26146996

[34] Liu L., Xiong Y., Gao H. et al. (2018) dbAMEPNI: a database of alanine mutagenic effects for protein–nucleic acid interactions. *Database*, 2018, 1–7. 10.1093/database/bay034 PMC5887268 29688380

